# Efficacy and safety of neoadjuvant imatinib therapy for patients with locally advanced rectal gastrointestinal stromal tumors: A multi-center cohort study

**DOI:** 10.3389/fphar.2022.950101

**Published:** 2022-09-27

**Authors:** Weihao Li, Xinyue Li, Kun Yu, Binyi Xiao, Jianhong Peng, Rongxin Zhang, Lingfang Zhang, Kun Wang, Zhizhong Pan, Cong Li, Xiaojun Wu

**Affiliations:** ^1^ State Key Laboratory of Oncology in South China, Collaborative Innovation Center for Cancer Medicine, Department of Colorectal Surgery, Sun Yat-sen University Cancer Center, Guangzhou, China; ^2^ Department of Colorectal Cancer Surgery, Yunnan Cancer Hospital, The Third Affiliated Hospital of Kunming Medical University, Kunming, China

**Keywords:** gastrointestinal stromal tumors, rectum, neoadjuvant imatinib therapy, surgical outcomes, prognosis

## Abstract

**Background:** Several issues on neoadjuvant imatinib therapy remain controversial despite its widespread application for rectal gastrointestinal stromal tumors (GIST). We aimed to describe the clinicopathological characteristics of this specific population, and compare the surgical and oncologic outcomes between patients with or without neoadjuvant imatinib therapy.

**Patients and methods:** A cohort of 58 consecutive locally advanced rectal GIST patients receiving surgical treatment between January 2007 and July 2019 at Sun Yat-sen University Cancer Center and Yunnan Cancer Hospital was retrospectively analyzed. Recurrence-free survival (RFS) and overall survival (OS) were estimated using Kaplan-Meier method.

**Results:** There were 33 (56.9%) patients who received neoadjuvant imatinib therapy. Among them, 20 (60.6%) patients had partial response (PR) as their best response, 11 (33.3%) patients had stable disease (SD), and 2 (6.1%) patients had progressive disease (PD). The median tumor size reduced from 5.2 to 4.0 cm after treatment (*p* < 0.001), and an attained “maximal response” was primarily achieved (32/33) on the 12th month after treatment. The most common adverse event was anemia. There were 27 adverse events occurred, most of which were grade 1 (19/27). With respect to intraoperative and postoperative surgical outcomes, no significant difference was found between patients with or without neoadjuvant Imatinib therapy except that patients with neoadjuvant treatment had a significant higher rate of preventive ileostomy (*p* = 0.004). Patients received neoadjuvant treatment had a superior 2-years RFS outcome than those without, though the difference was no significant (91.7% vs. 78.9%, *p* = 0.203). There were no significant differences in the 2-years OS rates (95.2% vs. 91.3%, *p* = 0.441).

**Conclusion:** Neoadjuvant imatinib therapy is an effective and safe treatment for locally advanced rectal GISTs. Further studies are warranted to validate the long-term prognostic benefit for patients with rectal GISTs receiving neoadjuvant imatinib therapy.

## Introduction

Gastrointestinal stromal tumors (GIST) are the most common mesenchymal malignancies of the digestive tract, originating from the interstitial cells of Cajal (Miettinen et al., 2005). Rectal GIST is a rare entity, accounting for approximately 5% of all GISTs (Tielen et al., 2013; Ulanja et al., 2019). Despite the low incidence, patients with rectal GIST have a worse prognosis than those occurring elsewhere in digestive tract as reported (Bischof et al., 2015).

Up to 90% of GISTs have gain-of-function mutations in either *c-kit* or platelet derived growth factor receptor *α* (*PDGFRA*) receptor tyrosine kinases (Rubin et al., 2001; Heinrich et al., 2003). Since the introduction of imatinib (IM), a tyrosine kinase inhibitor that blocks the kinase activity of both *c-kit* and *PDGFRA*, it has been proven to be an effective therapy and widely used in the treatment of GISTs (Dematteo et al., 2009; Joensuu et al., 2013). Although surgery with R0 margin is the only curative treatment for non-metastatic GISTs, owing to the complexity and narrow space of the anatomical structure and often large in size of rectal GIST, surgical treatments for GISTs in this region are challenging. Moreover, lymph node dissection is usually unnecessary for GIST because its metastasis is rare, which means that transanal endoscopic microsurgery (TME), a minimally invasive with less complications and better sphincter preservation, is feasible for rectal GIST with appropriate size. Hence, to shrink rectal GIST size and increase the possibility of complete resection and anal preservation, neoadjuvant imatinib therapy has been investigated in an attempt to provide a new approach for the treatment of rectal GISTs in last decades, and several studies (Fujimoto et al., 2014; Kyo et al., 2016; Pai et al., 2016; Cavnar et al., 2017; Kaneko et al., 2017; Liu et al., 2021; Yang et al., 2021) have reported its efficacy and safety.

Though the combination of neoadjuvant imatinib therapy and surgical treatment has been recommended by both Current National Comprehensive Cancer Network (NCCN) (von Mehren et al., 2018) and European Society of Medical Oncology (ESMO) (Casali et al., 2022) guidelines, several issues remain unclear, including optimal duration of neoadjuvant treatment, appropriate surgical approach and long-term survival benefit. Therefore, in current study, we aimed to describe the clinicopathological characteristics and prognosis of locally advanced rectal GIST patients receiving surgical treatment in our center with or without neoadjuvant imatinib therapy, and compare the surgical and oncologic outcomes between these two populations.

## Patients and methods

### Patient selection

We obtained medical records of 1,626 consecutive patients diagnosed with GIST between January 2007 and July 2019 at Sun Yat-sen University Cancer Center (SYSUCC) and Yunnan Cancer Hospital (YNCH). The inclusion criteria were as follows: 1) histologically confirmed rectal GIST; 2) complete record of demography, clinicopathological characteristics, the preoperative and postoperative targeted therapy and surgery; 3) at least a 3-months postoperative follow-up; 4) no other anti-tumor therapy was received before surgery expect imatinib therapy; 5) no distant metastatic disease before surgery or accompanying other malignant tumors. Finally, 58 rectal GISTs were included (45 patients from SYSUCC and 13 patients from YNCH). Patient demographics, tumor characteristics, surgical records, preoperative and postoperative adjuvant therapy records were carefully reviewed. The present study was performed according to the ethical standards of the World Medical Association Declaration of Helsinki and approved by the Institutional Review Board and Independent Ethics Committees of Sun Yat-sen University Cancer Center (approval number: B2021-457-01). The informed consent requirement was waived based on the nature of this retrospective study, in which patient data were kept confidential.

### Treatments and measurements

The criteria for the administration of neoadjuvant imatinib treatment were as follow: 1) difficulty in achieving R0 resection, 2) resectable tumor while combined organ resection is needed, 3) expected difficulty in achieving anal preservation, or 4) high risk of tumor rupture. The treatment strategy for each patient was determined according to the final agreement of the multidisciplinary team (MDT). Most patients with neoadjuvant treatment received oral imatinib 400 mg daily. Only one patient with exon nine mutation received imatinib 600 mg daily. Imatinib was continued until a “maximal response” was attained, which was defined as the non detection of further regression or decline to SD in two consecutive CT/MRI scans, or if the surgeon considered a radical resection possible, which was determined by attending physician.

Response evaluation during therapy was performed every 3 months using High-resolution magnetic resonance imaging (MRI) and/or contrast-enhanced computed tomography (CT) scans that were reviewed by a trained radiologist in one of the expertise centers following Choi criteria (Choi, 2008). The best response was classified as a complete response (CR), partial response (PR), stable disease (SD), or progressive disease (PD). The intensity of the adverse events during chemotherapy was graded according to the National Cancer Institute Common Terminology Criteria for Adverse Events (NCI CTCAE), version 5.0. Surgery procedure was determined by the physician, mainly based on tumor location and size, including Transanal Endoscopic Microsurgery (TEM), Low Anterior Resection (LAR), Abdominoperineal Resection (APR). Postoperative complications were graded according to the Clavien-Dindo classification (Clavien et al., 2009). Risk classification was evaluated according to the modified National Institutes of Health (mNIH) consensus criteria (Fletcher et al., 2002; Rutkowski et al., 2011).

### Follow-up

The primary endpoint was 2-years recurrence-free survival (RFS) and 2-years overall survival (OS). RFS was defined as the interval from surgery to disease recurrence, death, or the last follow-up. OS was defined as the interval from the date of surgery until the death of any cause or the last follow-up; patients without any event (metastasis or death) at the last follow-up date were regarded as random censoring. All patients were observed through subsequent visits every 3 months for 2 years and then semiannually until 3 years after surgery. Physical examination, blood tests, abdominal ultrasonography, and chest X-ray were conducted every 3 months postoperatively. Chest/abdominal/pelvic computed tomography (CT) and colonoscopy were performed annually. Response evaluation during systemic therapy was performed every 3, 6, or 12 months using CT scans.

### Statistical analysis

All statistical analyses were performed using IBM SPSS statistics software, version 23.0 (IBM Corp., Armonk, NY, United States). Continuous variables are described as means ± SD and were analyzed using the Student’s *t*-test or Mann-Whitney U test. Categorical variables are given as percentages and were compared using the Chi-square or Fisher’s exact test when appropriate. The OS and RFS rates were estimated with the Kaplan-Meier method, and the differences between groups were then assessed with the log-rank test. All of the statistical tests were two-sided; *p* < 0.05 was considered significant.

## Results

### Patient characteristics

**FIGURE 1:**
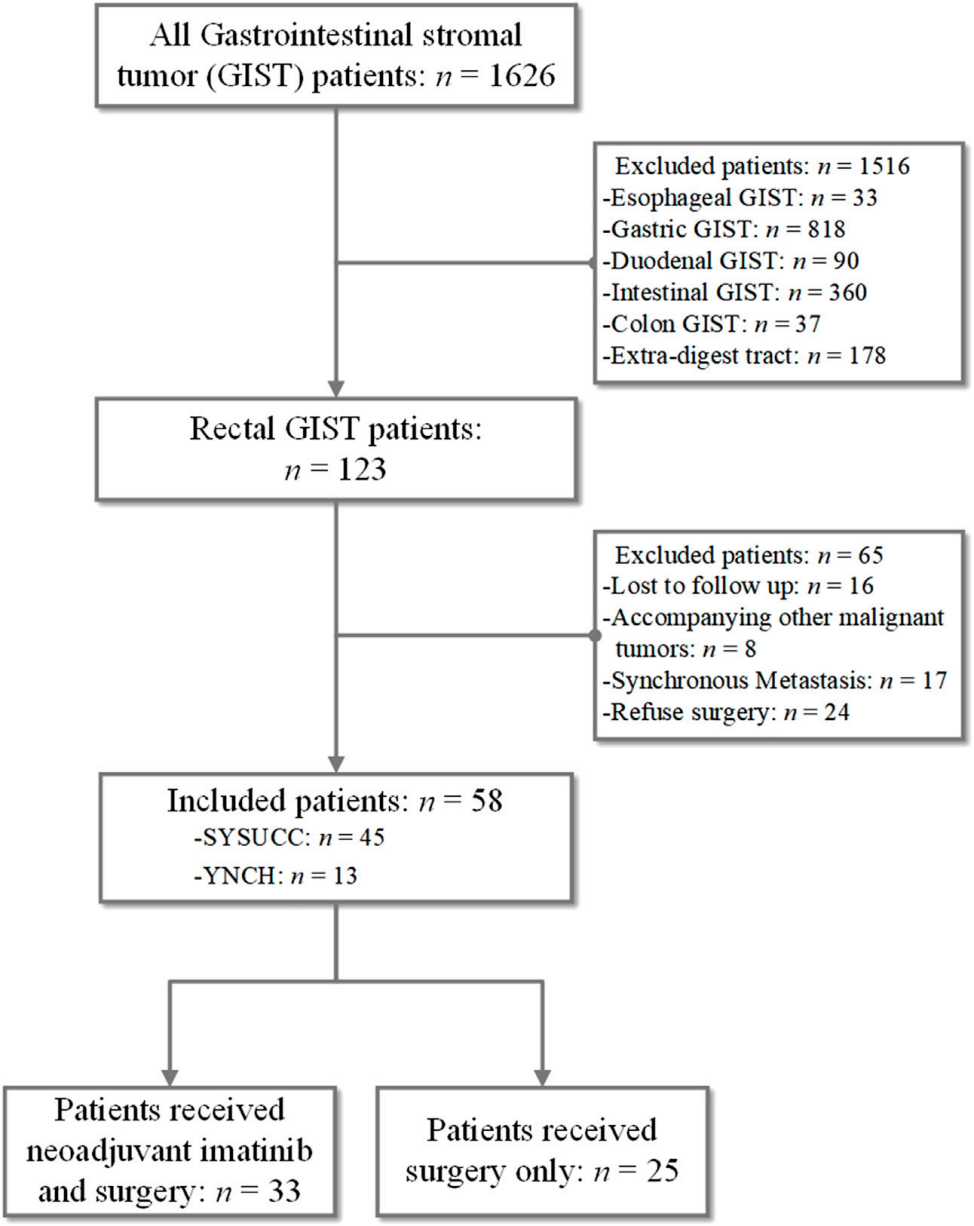
Flow chart of patient inclusion.

As shown in Figure 1, 58 patients with rectal GIST were included, accounting for 33 patients who received neoadjuvant imatinib therapy and surgery and 25 patients received surgery only. As shown in Table 1, for the whole rectal GIST, the most common symptoms were hematochezia (24.1%) and there were 20 (34.5%) patients without presenting symptoms at diagnosis. There were 36 (62.1%) patients receiving TEM surgery procedures and the R0, R1 resection rate was 81.0%, 19.0%, respectively. No R2 resection was observed in these patients. The positive rates of CD117, DOG-1 and CD34 were 100%, 86.2%, and 91.4%, respectively. There were 46 (79.3%) cases with mitotic count ≤5/50 HPF and 12 (20.7%) cases >5/50HPF. According to the mNIH risk classification, there were four patients (6.9%) with very low risk, 13 patients (22.4%) with low risk, four patients (6.9%) with intermediate risk, and 37 patients (63.8%) with high risk. Seventeen (29.3%) patients received adjuvant imatinib therapy after surgery.

**TABLE 1:** Characteristics of the total patients in the study.

Variables	All patients (*n* = 58)
Median age (range)—years	55 (22-82)
Gender—no. (%)	
Male	37 (63.8)
Female	21 (36.2)
Median distance of the tumor from anal verge (range)—cm	3.0 (1.0–8.0)
Median baseline tumor size (range)—cm	5.2 (1.0–14.3)
Median preoperative tumor size (range)—cm	4.0 (1.0–14.0)
Presenting symptoms—no. (%)	
Yes	38 (65.5)
Hematochezia	14 (24.1)
Abdominal pain	7 (12.1)
Constipation	11 (19.0)
Diarrhea	5 (8.6)
Anal pain	5 (8.6)
No	20 (34.5)
Median neoadjuvant treatment duration (range)—months	12.0 (3.1–29.5)
Surgical procedure—no. (%)	
TEM	36 (62.1)
LAR	12 (20.7)
APR	10 (17.2)
Margin—no. (%)	
R0	47 (81.0)
R1	11 (19.0)
IHC—no. (%)	
CD117 (+)	58 (100.0)
Dog-1 (+)	50 (86.2)
CD34 (+)	53 (91.4)
Mitotic count (postoperative)—no. (%)	
≤5/50 HPF	46 (79.3)
>5/50 HPF	12 (20.7)
Gene mutation type—no. (%)	
Exon 11	25 (43.1)
Exon 9	1 (1.7)
Others	0
Unavailable	32 (55.2)
mNIH risk stratification—no. (%)	
Very low	4 (6.9)
Low	13 (22.4)
Intermediate	4 (6.9)
High	37 (63.8)
Adjuvant IM—no. (%)	
Yes	17 (29.3)
No	41 (70.7)

Abbreviations: TEM, transanal endoscopic microsurgery; LAR, low anterior resection; APR, abdominoperineal resection; HPF, high-power field; IHC, immunohistochemistry; mNIH, modified National Institutes of Health; IM, imatinib.

As shown in Table 2, although the neoadjuvant imatinib treatment group presented a significantly higher proportion of baseline larger tumors than those in the surgery alone group (75.8% vs. 20.0%; *p* = 0.001), there were no significant differences between the two groups in terms of preoperative tumor size (*p* = 0.087). A significantly higher proportion of low mitotic count was found in the neoadjuvant group (93.9% vs. 60.0%; *p* = 0.001), while a higher high risk mNIH risk classification proportion was found in the neoadjuvant group than that in the surgery alone group (72.7% vs. 52.0%; *p* = 0.011). There were more patients receiving postoperative adjuvant IM therapy in the neoadjuvant group (81.8% vs. 56.0%; *p* = 0.032). There were no significant differences between the two group in terms of age, gender, distance of the tumor from anal verge, presenting symptoms, tumor rupture, mitotic count, gene mutation type distribution, mNIH risk grading. More details were shown in Table 2.

**TABLE 2:** Characteristics of the 58 study patients with locally advanced rectal GIST grouped by whether having received neoadjuvant Imatinib therapy.

Variables	Neoadjuvant group (*n* = 33, %)	Surgery alone group (*n* = 25, %)	*p* value
Age (years)			0.792
≤60	20 (60.6)	16 (64.0)	
>60	13 (39.4)	9 (36.0)	
Gender			0.282
Male	23 (69.7)	14 (56.0)	
Female	10 (30.3)	11 (44.0)	
Distance of the tumor from anal verge (cm)			0.562
≤2	13 (39.4)	8 (32.0)	
>2	20 (60.6)	17 (68.0)	
Baseline tumor size (cm)			**0.001**
≤5	8 (24.2)	20 (80.0)	
>5	25 (75.8)	5 (20.0)	
Preoperative tumor size (cm)			0.087
≤4	15 (45.5)	17 (68.0)	
>4	18 (54.5)	8 (32.0)	
Presenting symptoms			0.984
Yes	22 (66.7)	16 (64.0)	
Bleeding	8 (24.2)	6 (24.0)	
Abdominal pain	5 (15.2)	2 (8.0)	
Constipation	6 (18.1)	5 (20.0)	
Diarrohea	3 (9.1)	2 (8.0)	
Anal pain	2 (6.1)	3 (12.0)	
No	11 (33.3)	9 (36.0)	
Tumor rupture			0.246
Yes	0	1 (4.0)	
No	33 (100.0)	24 (96.0)	
Mitotic count (postoperative) (/50 HPF)			**0.002**
≤5	31 (93.9)	15 (60.0)	
>5	2 (6.1)	10 (40.0)	
IHC			0.817
CD117 (+)	33 (100.0)	25 (100.0)	
Dog-1 (+)	28 (84.8)	22 (88.0)	
CD34 (+)	30 (90.9)	23 (92.0)	
Gene mutation type			0.167
Exon 11	13 (39.4)	12 (48.0)	
Exon 9	0	1 (4.0)	
Others	0	0	
Unavailable	20 (60.6)	12 (48.0)	
mNIH risk stratification			**0.011**
Very low	0	4 (16.0)	
Low	5 (15.2)	8 (32.0)	
Intermediate	4 (12.1)	0	
High	24 (72.7)	13 (52.0)	
Adjuvant IM			**0.032**
Yes	27 (81.8)	14 (56.0)	
No	6 (18.2)	11 (44.0)	

Abbreviations: HPF, high-power field; IHC, immunohistochemistry; mNIH, modified National Institutes of Health; IM, imatinib.

Bold values represents when the *p*-value result is less than 0.05.

### Neoadjuvant imatinib therapy

**FIGURE 2:**
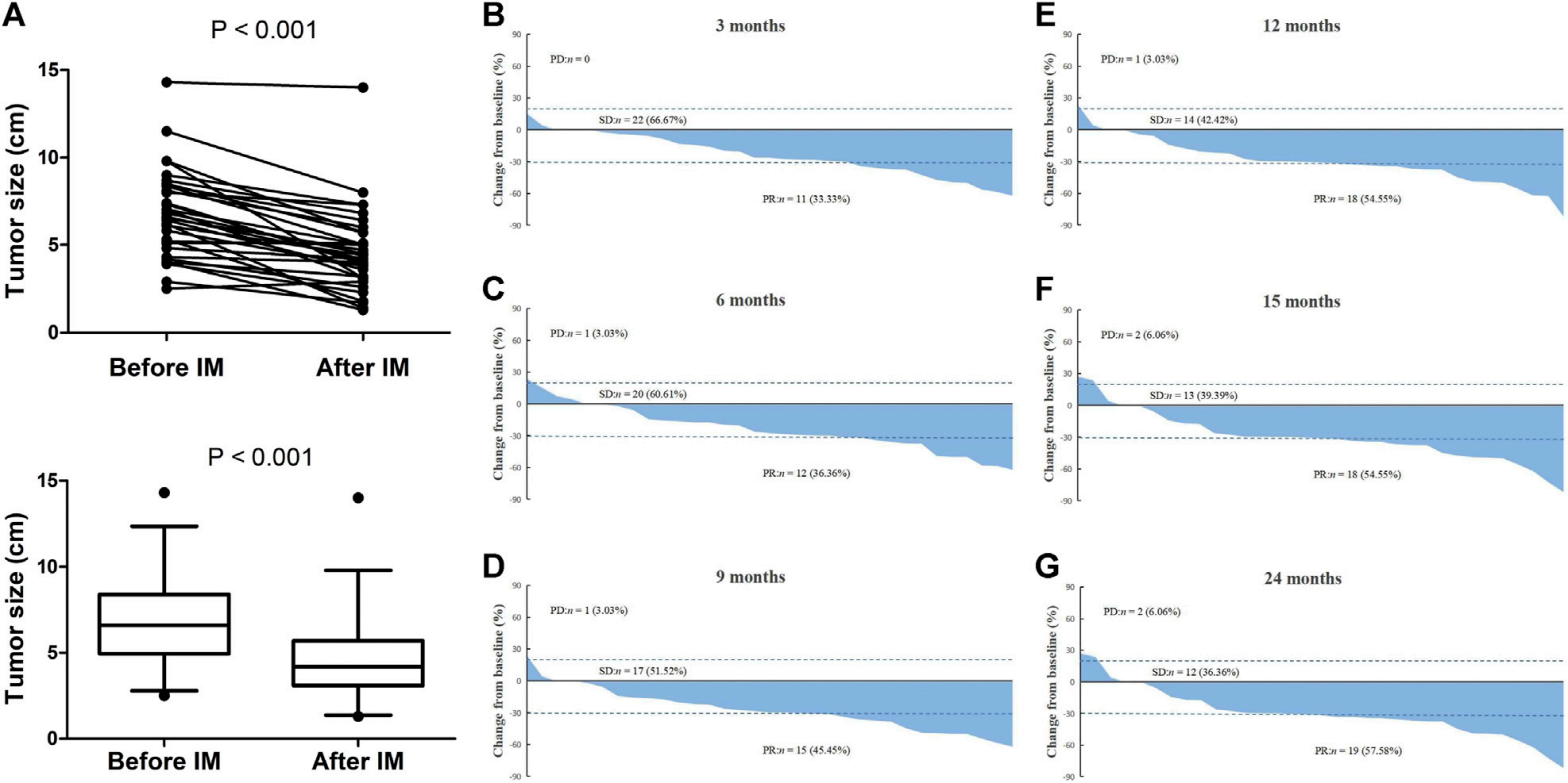
Treatment response evaluation of locally advanced rectal GIST patients with neoadjuvant imatinib therapy. **(A)** Comparison of tumor size before and after treatment. **(B–G)** Waterfall chart showing tendency of treatment response evaluation on the 3, 6, 9, 12, 15, 24 months after receiving neoadjuvant imatinib therapy. Note: If patient discontinued treatment at a specific time-point, the last time of treatment response evaluation would be used to present in all later waterfall charts. For example, a patient stopped treatment on the sixth month and the last one of treatment response evaluations was PR, we signed this patient as PR in the later ninth, 12th, 15th, and 24th month waterfall charts.

Eventually, there were 33 (56.9%) patients received neoadjuvant imatinib therapy. The median treatment duration was 12.0 (3.1–29.5) months. Among them, treatment duration of 8 (24.2%) patients were no more than 6 months and 25 (75.8%) patients were more than 6 months. As shown in Figure 2A, the median tumor size reduced from 5.2 to 4.0 cm after treatment (*p* < 0.001). Of the whole neoadjuvant imatinib treatemt cohort, 20 (60.6%) patients had PR as their best response, 11 (33.3%) patients had SD as their best response, and 2 (6.1%) patients had PD. No CR was observed in these patients. Figures 2B–G present the tendency of treatment response evaluation on the 3, 6, 9, 12, 15, 24 months after receiving imatinib therapy and we found that an attained “maximal response” was primarily achieved (32/33) on the 12th month.

The major adverse events during neoadjuvant imatinib therapy are presented in Table 3. No patient suffered from grade 4 adverse event. Totally three patients suffered from grade 3 adverse event but none was hospitalized for treatment. The most common adverse event was anemia. There were 28 adverse events that occurred in this cohort and most of them were grade 1 (20/28).

**TABLE 3:** Summary of neoadjuvant imatinib treatment-related adverse events.

Adverse events	Neoadjuvant group (*n* = 33, %)
Anemia	
Total	11 (33.3)
Grade 1	8 (24.2)
Grade 2	2 (6.1)
Grade 3	1 (3.0)
Grade 4	0
Leucopenia	
Total	6 (18.2)
Grade 1	3 (9.1)
Grade 2	2 (6.1)
Grade 3	1 (3.0)
Grade 4	0
Thrombocytopenia	
Total	2 (6.1)
Grade 1	2 (6.1)
Grade 2	0
Grade 3	0
Grade 4	0
Nausea/vomiting	
Total	1 (3.0)
Grade 1	1 (3.0)
Grade 2	0
Grade 3	0
Grade 4	0
Diarrhea	
Total	1 (3.0)
Grade 1	1 (3.0)
Grade 2	0
Grade 3	0
Grade 4	0
Edema	
Total	3 (9.1)
Grade 1	2 (6.1)
Grade 2	1 (3.0)
Grade 3	0
Grade 4	0
Hepatic disorder	
Total	2 (6.1)
Grade 1	1 (3.0)
Grade 2	0
Grade 3	1 (3.0)
Grade 4	0
Renal disorder	
Total	2 (6.1)
Grade 1	2 (6.1)
Grade 2	0
Grade 3	0
Grade 4	0

Notes: The listed grades of adverse events represent the maximal levels at any time.

### Surgical outcomes

Table 4 presented the comparison of surgical outcomes between patients whether having received neoadjuvant Imatinib therapy. For the selection of surgery procedures, there was no difference between the two groups (*p* = 0.089). However, patients in neoadjuvant group had a significant higher rate of preventive ileostomy than those in surgery alone group (27.3% vs. 0; *p* = 0.004). Between the two groups, the rates of R0 resection (*p* = 0.395), anus preservation (*p* = 0.412), and combined organ resection (*p* = 0.975) were similar. With respect to intraoperative and postoperative recovery outcomes, no significant difference was found between the two groups in terms of procedure time, intraoperative transfusion volume, intraoperative urine volume, estimated blood loss, blood transfusion, rate of 30-days postoperative complication, time to diet, length of stay after surgery, rate of 30-days postoperative mortality and rate of long-term postoperative complication.

**TABLE 4:** Comparison of surgical outcomes of the 58 patients with locally advanced rectal GIST grouped by whether having received neoadjuvant imatinib therapy.

Variables	Neoadjuvant group (*n* = 33, %)	Surgery alone group (*n* = 25, %)	*p* value
Surgical procedure			0.089
TEM	19 (57.6)	17 (68.0)	
LAR	10 (30.3)	3 (12.0)	
APR	4 (12.1)	5 (20.0)	
Procedure time (minutes)	170.36 ± 77.73	139.40 ± 91.61	0.181
Intraoperative transfusion volume (ml)	2,500 (1,500–3,300)	2000 (1,000–4,500)	0.426
Intraoperative urine volume (ml)	500 (200–2,000)	350 (100–2,100)	0.418
Estimated blood loss (ml)	111.73 ± 86.78	79.46 ± 36.69	0.527
Blood transfusion	1 (3.85)	0	0.975
Margin			0.395
R0	28 (84.8)	19 (76.0)	
R1	5 (15.2)	6 (24.0)	
Anus preservation			0.412
Yes	29 (87.9)	20 (80.0)	
No	4 (12.1)	5 (20.0)	
Preventive ileostomy			
Yes	9 (27.3)	0	**0.004**
No	24 (72.7)	25 (100)	
Combined organ resection	1 (3.85)	0	0.975
30-days postoperative complication			0.628
Anastomotic leakage	3 (9.1)	1 (4.0)	
Anastomotic bleeding	0	1 (4.0)	
Intestinal obstruction	1 (3.0)	0	
Delay wound healing	2 (6.1)	1 (4.0)	
Pelvic hemorrhage	1 (3.0)	1 (4.0)	
Time to diet (days)	2.54 ± 1.66	2.00 ± 1.45	0.265
LOS after surgery (days)	6.23 ± 2.79	6.00 ± 3.00	0.792
30-days postoperative mortality	0	0	1.000
Long-term postoperative complication			0.524
Defecated dysfunction	15 (45.5)	8 (32.0)	
Urinary dysfunction	1 (3.0)	0	
Sexual dysfunction	4 (12.1)	4 (16.0)	

Abbreviations: TEM, transanal endoscopic microsurgery; LAR, low anterior resection; APR, abdominoperineal resection; LOS, length of stay.

Bold values represents when the *p*-value result is less than 0.05.

### Survival analysis

**FIGURE 3:**
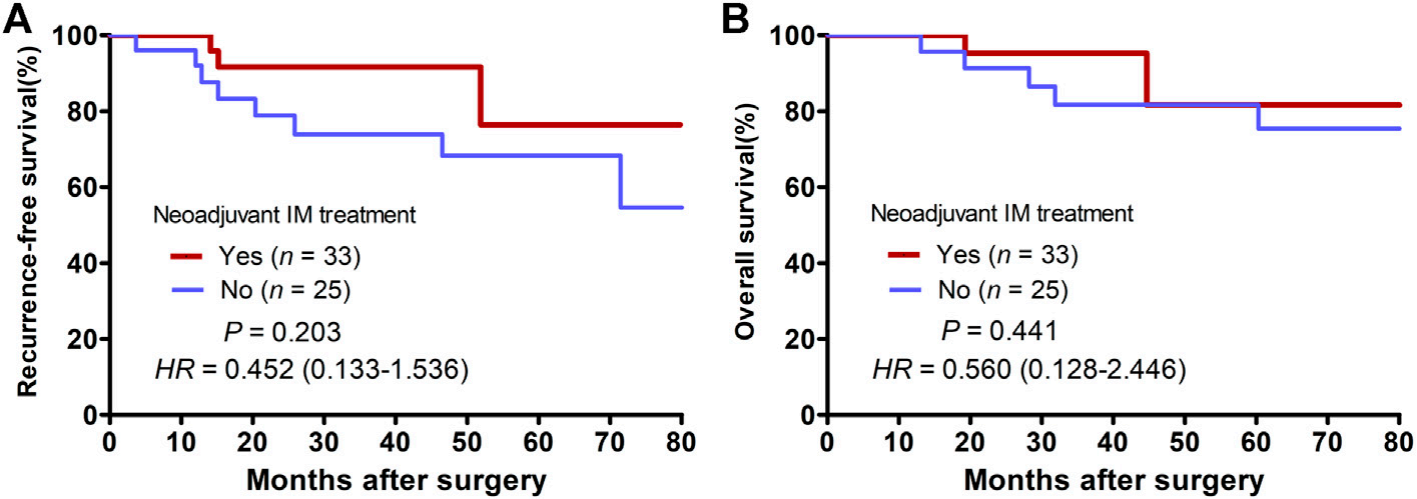
Kaplan-Meier curves of patients with locally advanced rectal GIST grouped by whether having received neoadjuvant Imatinib therapy. **(A)** Comparison of recurrence-free survival between the neoadjuvant group and the surgery alone group. **(B)** Comparison of overall survival between the neoadjuvant group and the surgery alone group.

The last follow-up visit was in July 2021. During the median follow-up time of 24.1 (6.4–172.3) months, 11 cases had postoperative recurrence, including 7 cases of local recurrence, 3 cases of liver metastasis, 1 case of vertebra metastasis. And finally eight patients died of cancer. Among them, only three patients received neoadjuvant imatinib therapy and seven patients received adjuvant imatinib therapy after surgery. According to mNIH risk classification, six patients were high risk. More details about postoperative recurrence cases can be found in Table 5. Of the entire cohort, the 2-years RFS and OS rate were 82.5% and 93.3%, respectively. The 2-years RFS rates were better in the neoadjuvant group than in the surgery alone group, while the difference was no significant (91.7% vs. 78.9%, *p* = 0.203, Figure 3A). There were no significant differences in the 2-years OS rates between the two groups (95.2% vs. 91.3%, *p* = 0.441, Figure 3B).

**TABLE 5:** Summary of postoperative recurrence cases.

Order	Sex	Age (years)	DAV (cm)	Baseline tumor size (cm)	Recurrence situation	Time to Recurrence (months)	Survival status	Neoadjuvant IM	Surgical procedure	Margin	Gene mutation type	mNIH risk stratification	Adjuvant IM
1	Female	58	4	8.4	Local recurrence	15.19	Death	Yes	LAR	R1	Unavailable	High risk	Yes
2	Female	50	3	5.0	Local recurrence	14.10	Death	Yes	APR	R0	Unavailable	High risk	Yes
3	Male	69	5	6.5	Local recurrence	51.88	Alive	Yes	APR	R0	C-Kit Exon 11	Intermediate risk	Yes
4	Female	63	2	4.4	Vertebra metastasis	15.16	Death	No	TEM	R0	C-Kit Exon 11	Low risk	Yes
5	Male	58	2	4.0	Local recurrence	20.38	Alive	No	TEM	R1	Unavailable	Low risk	No
6	Female	63	3	3.5	Liver metastasis	12.85	Death	No	TEM	R0	C-Kit Exon 11	High risk	Yes
7	Male	62	2	7.6	Liver metastasis	25.84	Death	No	APR	R0	C-Kit Exon 9	High risk	No
8	Female	59	2	3.0	Liver metastasis	71.47	Death	No	TEM	R1	C-Kit Exon 11	High risk	Yes
9	Male	50	3	7.9	Local recurrence	12.00	Death	No	APR	R0	C-Kit Exon 11	High risk	Yes
10	Male	64	6	4.0	Local recurrence	1.66	Death	No	TEM	R0	C-Kit Exon 11	Low risk	No
11	Male	37	6	5.4	Local recurrence	46.49	Alive	No	LAR	R0	Unavailable	Low risk	No

Abbreviations: DAV, inferior tumor margin from the anal verge; mNIH, modified National Institutes of Health; IM, imatinib; TEM, transanal endoscopic microsurgery; LAR, low anterior resection; APR, abdominoperineal resection.

## Discussion

In this retrospective study, we investigated the clinicopathological characteristics of locally advanced rectal GIST patients receiving surgery with or without neoadjuvant imatinib therapy and compared the surgical and oncologic outcomes between these two groups. Here, we found that for locally advanced rectal GISTs with a relatively large tumor size, neoadjuvant imatinib therapy is an effective and safe treatment to reduce tumor size, and achieves a similar rates of complete resection and anal preservation without increasing intraoperative or postoperative complications risk. Moreover, we found that the 2-years RFS rates were better in the neoadjuvant group than in the surgery alone group, though the difference was no significant. The results provided further evidence supporting the administration of neoadjuvant imatinib therapy for locally advanced rectal GIST patients.

Due to natural growth characteristics of rectal GIST, including exophytic growth and middle-low rectum location (extraperitoneal), rectal GISTs are often large in size and without presenting symptoms at first diagnosis (Jakob et al., 2013; Liu et al., 2014). Recently, a Chinese multi-institutional study (Yang et al., 2021) reported that 10% of rectal GIST patients had tumor exceeding 10 cm and 91% of cases occurred in the lower rectum at diagnosis, which brought a huge challenge to achieve complete tumor resection and preservation of anal and organs. And after neoadjuvant treatment, the median tumor size of rectal GIST patients reduced significantly from 5.0 to 4.0 cm, with a PR rate of 75%. A large European cohort (NS et al., 2020) has reported that median tumor downsizing rate with neoadjuvant imatinib therapy in rectal GISTs was 33%. In current study, median tumor size reduced from 5.2 to 4.0 cm, with a PR rate of 60.6% and a disease control rate (DCR) of 93.9%, which was consistent with previous studies (Machlenkin et al., 2011; Jakob et al., 2013; Bednarski et al., 2014; Huynh et al., 2014; NS et al., 2020). Tumor downsizing after neoadjuvant treatment enables patients with large tumors to receive less invasive surgery with a safe margin and function preservation, and reduce the risk of tumor rupture. Neoadjuvant treatment did not increase the risk of intraoperative and postoperative complications but probably at the cost of elevated preventive ileostomy rate.

The optimal duration of neoadjuvant imatinib therapy is still controversial. In current clinical practice, the consensus is that imatinib should be continued until a “maximal response” was attained, which was defined by MDT as previously mentioned. The NCCN guidelines (von Mehren et al., 2018) noted that the “maximal response” may require neoadjuvant treatment for 6 months or even more. Previous researches (Bednarski et al., 2014; Yang et al., 2021) have found that the duration of neoadjuvant therapy more than 12 months was associated with a higher risk of recurrence, which may contribute to patients’ inherent worse tumor biology and lower treatment response rate, meaning a relatively inferior prognosis. Another problem is that long lasting imatinib therapy may probably induce the development of secondary resistance related to additional *c-kit* mutations. We explored the tendency of treatment response evaluation and found that an attained “maximal response” was primarily achieved (32/33) on the 12th month. One patient even had PD on the 15th month. This finding need to be validated further in a prospective, multicenter clinical trial.

Whether neoadjuvant imatinib therapy brings prognosis benefits is also controversial. Hawkins et al. (2017) found that preoperative chemotherapy may result in improved survival for large anorectal GISTs treated with radical resection (5-years OS with chemotherapy 79.2% vs. no chemotherapy 51.2%; *p* = 0.03). Jakob et al. (2013) found that perioperative imatinib was associated with improved local disease-free, disease-free, and overall survival (*p* < 0.01, *p* < 0.01, and *p* = 0.03, respectively). Patients with preoperative imatinib had a significantly higher rate of R0 resections and local disease-free survival was significantly improved by negative resection margins (*p* < 0.01). Cavnar et al. (2021) revealed that in primary GIST patients undergoing neoadjuvant imatinib therapy, High tumor mitotic rate and incomplete resection following neoadjuvant imatinib treatemt were associated with poor outcomes, while adjuvant imatinib therapy was associated with prolonged survival. Our study found a prolonged RFS in patients received neoadjuvant imatinib therapy, though the difference was no significant. Although there were no significant differences between the two groups in terms of R0/R1 resection rate, we can find a tendency of higher R0 resection rate in neoadjuvant group (84.8% vs. 76.0%, *p* = 0.395). And a significantly higher proportion of low mitotic count was found in the neoadjuvant group, which was a positive prognostic factor as previously report (Cavnar et al., 2021). At the mean time, there were more patients receiving postoperative adjuvant IM therapy in the neoadjuvant group (81.8% vs. 56.0%; *p* = 0.032), which may bring prognosis benefit, resulting in bias in the comparison. Interestingly, patients in surgery alone group inclined to have more distance recurrence (4/8), while none in neoadjuvant group, which may credit to the effect of early-stage systemic therapy. In fact, a higher high risk mNIH risk classification proportion was found in the neoadjuvant group. Due to the change of mitotic counts and tumor size, current risk classification system might not be suitable for GIST patients receiving neoadjuvant treatment.

Several limitations should be acknowledged in the present study. First, this retrospective study included an uncontrolled methodology and a limited number of patients from a single cohort. The findings need to be validated in a prospective, multicenter clinical trial. Second, gene mutation status was linked to different recurrence risks and survival outcomes in GIST, while the gene mutation status information was imperfect in current study. Third, the short follow-up duration was only sufficient for patients to evaluate 2-years survival outcomes, which may have led to misestimation of the effect of neoadjuvant imatinib therapy on RFS and OS. Last but no least, the NCCN guideline for GIST suggested that risk classification based on nuclear mitotic index was not proper for patients receiving neoadjuvant because of inaccuracy due to the change of mitotic counts and tumor size. As a result, current risk classification system might not be suitable for GIST patients receiving neoadjuvant treatment.

## Conclusion

Neoadjuvant imatinib therapy is an effective and safe treatment to reduce tumor size for locally advanced rectal GISTs, and achieves a similar rates of complete resection and anal preservation without increasing intraoperative or postoperative complications risk. The optimal duration of neoadjuvant imatinib therapy might be 12 months. Further studies are warranted to validate the long-term prognostic benefit for patients with rectal GISTs receiving neoadjuvant imatinib therapy.

## Data Availability

The datasets used and analyzed during the current study are available from the corresponding author on reasonable request. The authenticity of this article has been validated by uploading the key raw data onto the Research Data Deposit public platform (www.researchdata.org.cn, RDDA2022754963).
